# Comparison of the Influence of Bisphenol A and Bisphenol S on the Enteric Nervous System of the Mouse Jejunum

**DOI:** 10.3390/ijms25136941

**Published:** 2024-06-25

**Authors:** Krystyna Makowska, Sławomir Gonkowski

**Affiliations:** 1 Department of Clinical Diagnostics, Faculty of Veterinary Medicine, University of Warmia and Mazury in Olsztyn, Oczapowskiego 14, 10-957 Olsztyn, Poland; 2 Department of Clinical Physiology, Faculty of Veterinary Medicine, University of Warmia and Mazury in Olsztyn, Oczapowskiego 13, 10-957 Olsztyn, Poland; slawomir.gonkowski@uwm.edu.pl

**Keywords:** mice, bisphenol A, bisphenol S, enteric nervous system, immunofluorescence studies, neurotransmitters

## Abstract

Bisphenols are dangerous endocrine disruptors that pollute the environment. Due to their chemical properties, they are globally used to produce plastics. Structural similarities to oestrogen allow bisphenols to bind to oestrogen receptors and affect internal body systems. Most commonly used in the plastic industry is bisphenol A (BPA), which also has negative effects on the nervous, immune, endocrine, and cardiovascular systems. A popular analogue of BPA-bisphenol S (BPS) also seems to have harmful effects similar to BPA on living organisms. Therefore, with the use of double immunofluorescence labelling, this study aimed to compare the effect of BPA and BPS on the enteric nervous system (ENS) in mouse jejunum. The study showed that both studied toxins impact the number of nerve cells immunoreactive to substance P (SP), galanin (GAL), vasoactive intestinal polypeptide (VIP), the neuronal isoform of nitric oxide synthase (nNOS), and vesicular acetylcholine transporter (VAChT). The observed changes were similar in the case of both tested bisphenols. However, the influence of BPA showed stronger changes in neurochemical coding. The results also showed that long-term exposure to BPS significantly affects the ENS.

## 1. Introduction

Human and animal exposure to environmental pollutants has significantly increased with the development of global industry. Endocrine disruptors like bisphenol A (BPA) are particularly dangerous. BPA is widely used in the production of plastic because of its chemical properties, including high endurance over a broad range of temperatures (−40 to 145 °C) and hardness [1].

However, due to its similarities to oestrogen, BPA can bind to oestrogen receptors and affect internal body systems [2]. In previous studies, exposure to BPA has been linked to obesity, thyroid malfunction, and lipid and glucose homeostasis alterations [3]. Moreover, BPA has also been documented to have negative effects on female and male reproductive function and the potential to interact with DNA even at low doses. This chemical may also interfere with cell division, leading to disorders including carcinogenesis [4]. As a result, BPA has been considered one of the most prevalent and harmful endocrine disruptors, which, even in relatively low doses, negatively affects living organisms [5]. Therefore, its use in industry has been restricted, and many countries have decided to withdraw BPA from the production of plastic equipment for babies [6,7,8]. Instead of bisphenol A, its analogues are increasingly used in the production of plastics. One of the most commonly used BPA analogues is bisphenol S (BPS).

When introduced, BPS was considered a harmless replacement for BPA. However, with increasing research, the knowledge about its harmful and similar BPA effects on living organisms has expanded [9,10]. It is known that BPS, like BPA, can impact the endocrine system, resulting in disturbances in reproduction, obesity, hypertension, and other disorders [11,12,13]. Moreover, this substance can cause carcinogenic effects, as well as neuro-, cyto-, and genotoxicity [10,14]. Furthermore, it has been mentioned that the oestrogenic activity of BPS may be even more advanced than that caused by BPA [9].

Despite the amount of research concerning the influence of bisphenols on living organisms, their impact on the gastrointestinal (GI) tract and the enteric nervous system (ENS) is still not fully known.

The organisation of the ENS, which regulates most functions of the GI tract and is localised in the wall of the oesophagus, stomach, and intestine, depends on the segment of the GI tract and the animal species. In the wall of the rodent small intestine, it is formed from two ganglionated plexuses—the submucous plexus (SmP), which is located in the submucosal layer in close proximity to the mucosa, and the myenteric plexus (MP), situated between the circular and longitudinal muscle layers [15].

Changes in the enteric neurons under physiological and pathological factors can be morphological, electrophysiological, or functional. This modification ability is known as the plasticity of neurons and may be an organism’s first adaptive or protective reaction to disorders of homeostasis caused by pathological and toxic stimuli [16]. The most visible changes in the ENS concern the neurochemical characterisation of neurons, i.e., the ability to produce and secrete active substances performing functions of neurotransmitters or neuromodulators [16,17,18]. Until now, several tens of active substances have been described in the ENS [19]. The most important of these are the following: acetylcholine, vasoactive intestinal polypeptide (VIP), nitric oxide, substance P (SP), and galanin (GAL) [17,20].

It should be underlined that choosing jejunum for the present investigation was not accidental. It is known that the small intestine is the longest section of the digestive tract, and intestinal villi also make it the largest absorptive surface in the whole gastrointestinal system. This is where the main part of digestion takes place, and from the wall of this organ, large amounts of mucus and intestinal juice are secreted [21]. Moreover, the small intestine is where bisphenols in food are absorbed and metabolised [22,23].

Considering the above, the present study aimed to investigate and compare for the first time the influence of BPA and BPS on the jejunal population of the enteric neurons containing selected active substances playing important functions in the regulation of GI tract activity, including VIP, the neuronal isoform of nitric oxide synthase (nNOS), which is a marker of nitrergic neurons, vesicular acetylcholine transporter (VAChT), which is a marker of cholinergic neurons, SP and GAL.

## 2. Results

During the present study, no clinical signs were observed in animals receiving BPA or BPS. There were also no differences in behaviour or amount of feed consumed. However, double-labelling immunohistochemistry revealed that all neuronal factors studied were present in neurons in both submucous and myenteric plexuses of mouse jejunum. Partial results obtained in the experiment are presented in Table 1.

**Figure 1:**
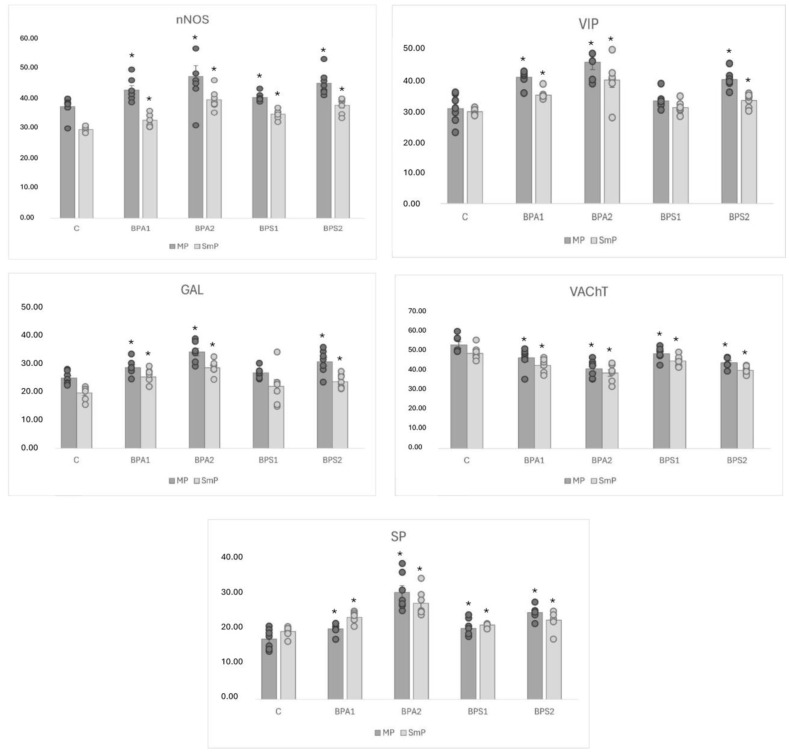
Graphical comparison of the percentage points of nerve cells immunoreactive to protein gene product (PGP 9.5), used here as a pan-neuronal marker, and other studied neurotransmitters: neuronal isoform of nitric oxide synthase (nNOS), vasoactive intestinal polypeptide (VIP), galanin (GAL), vesicular acetylcholine transporter (VAChT), and substance P (SP) in submucous (SmP) and myenteric plexus (MP) of mice jejunum with individual data points of every studied animal between physiological conditions (C) and experimental groups: after the administration of low (BPA1) and high (BPA2) doses of BPA and low (BPS1) and high (BPS2) doses of BPS. Statistically significant differences between the control and experimental groups are marked with *.

**Figure 2:**
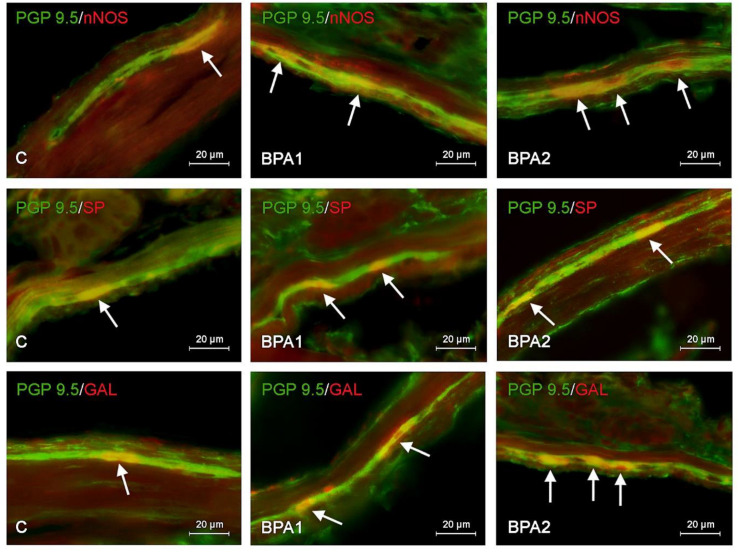
Nerve cells immunoreactive to protein gene product (PGP 9.5), used here as a pan-neuronal marker, and other studied neurotransmitters: neuronal isoform of nitric oxide synthase (nNOS), substance P (SP), and galanin (GAL) in myenteric plexus of mice jejunum under physiological conditions (C), after the administration of low (BPA1) and high (BPA2) doses of BPA. Neurons positive for selected substances are marked with arrows.

**Figure 3:**
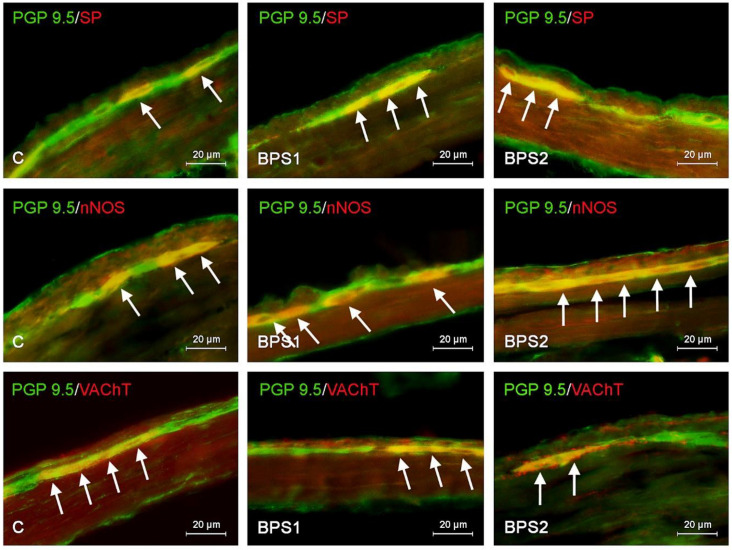
Nerve cells immunoreactive to protein gene product (PGP 9.5), used here as a pan-neuronal marker, and other studied neurotransmitters: substance P (SP), neuronal isoform of nitric oxide synthase (nNOS), and vesicular acetylcholine transporter (VAChT) in the myenteric plexus of mice jejunum under the physiological condition (C), after administration of low (BPS1) and high (BPS2) doses of BPS. Neurons positive for selected substances are marked with arrows.

In physiological conditions, the most numerous population consists of neurons containing VAChT. The percentage of such neurons amounted to 53.50 ± 1.63% and 49.11 ± 1.35% of all PGP 9.5-positive cells in the MP and SmP, respectively. A slightly smaller population (approximately one-third of cells immunoreactive to PGP 9.5) consisted of neurons containing nNOS and/or VIP. In particular, nNOS was found in 37.25 ± 1.28% of cells in the MP and 29.53 ± 0.37% in the SmP. In the case of VIP, these values amounted to 30.56 ± 1.77% and 29.59 ± 0.42% in the MP and SmP, respectively. Neurons immunoreactive to GAL and/or SP were less numerous. GAL was found in 25.54 ± 0.89% of all PGP 9.5-positive cells in the MP and 20.16 ± 0.92% in the SmP. In turn, the percentage of neurons immunoreactive to SP amounted to 17.16 ± 1.07% and 19.32 ± 0.53% in the MP and SmP, respectively (Table 2, Figure 1, Figure 2 and Figure 3).

During the present experiment, changes in the immunoreactivity of enteric neurons were observed in the case of both types of intramural plexuses after the administration of both bisphenols. Generally, those changes consisted of an increase in the number of cells containing nNOS, VIP, GAL, and/or SP with a simultaneous decrease in VAChT+ neurons. The observed changes depended on the type of bisphenol and its dose, and more visible fluctuations were noted in animals treated with BPA (Table 2, Figure 1, Figure 2 and Figure 3).

In the MP, the most visible BPA-induced changes were noted in the neurons that were immunoreactive to VIP. The percentage of such neurons in animals treated with a lower dose of BPA amounted to 40.66 ± 0.92% of all PGP 9.5-positive cells (increase by approx. 10 percentage points (pp) compared to the control animals, and in animals treated with a higher dose of BPA, the percentage reached 45.52 ± 2.39% (an increase of approx. 15 pp)). Slightly smaller changes were observed in the case of neurons containing nNOS and/or GAL. In animals of the BPA1 group, the percentage of nNOS-positive neurons was 42.74 ± 1.42% (an increase of approx. 5 pp), and in animals of the BPA2 group, it was 47.21 ± 3.67% (an increase of approx. 10 pp). For GAL-positive neurons, these values amounted to 29.31 ± 1.06% (an increase of approximately 4 pp) and 35.08 ± 1.36 (an increase of approximately 10 pp) in BPA1 and BPA2 groups, respectively. In the MP, BPA also caused an increase in the percentage of neurons containing SP to 20.00 ± 0.58% (an increase of approx. 3 pp) in the case of lower doses and to 30.38 ± 1.93% (an increase of approx. 13 pp) in the case of higher doses (Table 2, Figure 2).

In the SmP, the most visible BPA-induced changes are in the increase in the percentage of neuron-concerned cells immunoreactive to VIP and/or GAL. In the BPA1 group, the percentage of VIP-positive cells amounted to 34.98 ± 0.62% (an increase of approx. 5 pp in comparison to the control animals) and in the BPA2 group to 39.81 ± 2.43% (an increase of approx. 10 pp). In turn, a lower dose of BPA caused an increase in GAL-positive cells to 29.31 ± 1.06% of all PGP 9.5-positive cells (an increase of approx. 6 pp) and higher doses of BPA to 29.32 ± 0.96% (an increase of approx. 9 pp). In the SmP, BPA-induced increases in the percentage of neurons containing nNOS and/or SP were also noted. In animals of the BPA1 group, the percentage of nNOS-positive cells amounted to 32.65 ± 0.82% (an increase of approx. 3 pp), and in animals of the BPA2 group to 39.52 ± 1.29% (an increase of approx. 10 pp). In the case of SP-positive neurons, these values achieved 23.31 ± 0.55% (an increase of approx. 4 pp) and 27.28 ± 1.42% (an increase of approx. 8 pp), respectively.

Contrary to other neuronal populations, BPA caused a decrease in the percentage of neurons containing VAChT, both in the MP and SmP. In the MP, the percentage of such neurons amounted to 46.74 ± 1.98% of all PGP 9.5-positive cells (decrease by approx. 7 pp in the comparison of the control animals) in the BPA1 group and 41.10 ± 1.62% (decrease by approx. 10 pp) in BPA2 group. Similar changes were noted in the SmP, where the lower dose of BPA caused a decrease in the percentage of VAChT-positive neurons to 42.88 ± 1.27% (by approx. 7 pp) and higher doses of this substance to 38.94 ± 1.65% (by approx. 11 pp).

As mentioned above, changes noted under the impact of BPS were generally less visible. Both doses of BPS studied caused an increase in the percentage of cells immunoreactive to nNOS and/or SP, both in the MP and SmP. In the BPS1 group, the percentage of neurons containing nNOS amounted to 40.28 ± 0.53% in the MP (an increase of approx. 3 pp in comparison to the control animals) and 34.70 ± 0.58% in the SmP (an increase of approx. 5 pp). In the case of SP-positive neurons, these values achieved 20.15 ± 0.84% (an increase of approx. 3 pp) and 21.15 ± 0.53% (an increase of approx. 2 pp) in the MP and SmP, respectively. In animals treated with lower doses of BPS, statistically significant changes in the percentage of neurons immunoreactive to VIP and/or GAL compared to the control animal were not observed (Table 2, Figure 1 and Figure 3).

The higher doses of BPS caused an increase in the percentage of neurons containing nNOS, VIP, GAL, and/or SP in both types of enteric plexuses. In the MP, the most visible changes concerned neurons that were immunoreactive to VIP and nNOS. The percentage of VIP-positive neurons increased to 39.98 ± 1.06% of all PGP 9.5-positive cells (an increase of approx. 9 pp in comparison to the control animals), and the percentage of neurons immunoreactive to nNOS increased to 45.03 ± 1.54% (by approx. 8 pp). The percentage of neurons containing SP and/or GAL amounted to 24.70 ± 0.68% (an increase of approx. 7 pp) and 31.50 ± 1.64% (an increase of approx. 6 pp), respectively. Changes noted in the SmP under the impact of higher doses of BPS were less visible than those observed in the MP. The percentage of nNOS-positive neurons achieved 37.62 ± 0.99% (an increase of approx. 8 pp in comparison to the control animals), VIP-positive neurons 33.21 ± 0.8% (an increase of approx. 4 pp), GAL-positive neurons 24.27 ± 0.98% (an increase of approx. 4 pp), and SP-positive cells 22.50 ± 0.98% (an increase of approx. 3 pp) (Table 2, Figure 1 and Figure 3).

Moreover, similarly to BPA, BPS caused a decrease in the percentage of neurons containing VAChT. In animals treated with lower doses of BPA, the percentage of such neurons amounted to 49.00 ± 1.22% in the MP and 45.22 ± 1.01 in the SmP (in both types of the enteric plexuses, there is a decrease by approximately 4 pp in comparison to the control animals). In animals treated with higher doses of BPA, the changes were more visible. The percentage of VAChT-positive cells amounted 44.25 ± 1.01% and 40.36 ± 0.74% in the MP and SmP, respectively. These values were lower than those noted in the control animals by approximately 9 pp. The summarisation of the obtained results is presented in Table 1 and Table 2.

## 3. Discussion

The results obtained during the present experiment, showing relatively large populations of nNOS-, VIP-, GAL-, SP-, and/or VAChT-positive neurons in the intramural plexuses of jejunum, are consistent with previous studies concerning the ENS in different mammal species, including humans [24,25,26,27]. Therefore, the present results confirm the important roles of the factors mentioned above in regulating intestine functions. However, it should be highlighted that the ENS in mice jejunum is still not fully described in the scientific literature [28]. Obviously, the presence of the above-mentioned substances in this part of the nervous system in rodents has been the subject of many publications, but the particular populations of nerve cells immunoreactive to selected neuronal factors, especially when it comes to the submucous plexus, is an underexploited topic. Very often, in the case of this animal species, only the muscular plexus is the subject of research, although it is commonly known that neurons located in the submucous plexus also play a significant role, primarily in the regulation of adsorption and secretion of mucus and intestinal juice [29].

Moreover, the present study has shown a significant effect of BPA and BPS on the neurochemical characterisation of neurons in the ENS of the mice jejunum. In general, these observations are consistent with previous studies on the impact of toxic agents on the gastrointestinal tract and confirm the ability of intestinal neurons to change their neurochemical characteristics under the influence of toxic substances [30,31,32].

Both bisphenols studied have affected the population of nNOS-, GAL-, VIP-, SP-, and/or VAChT-positive neurons; however, the intensification of changes depended on the examined plexus and the type and dose of bisphenol.

BPA- and BPS-induced decrease in the number of cholinergic neurons was noted in the present study. Similar observations have been described in previous studies concerning the autonomic nervous system of other organs under the impact of BPA [33,34,35]. Moreover, similar changes have been previously noted as a result of various pathological and toxic stimuli in other parts of the gastrointestinal tract [30,31,32], which can imply blocking of the synthesis of acetylcholine during pathological conditions. This, in turn, suggests that acetylcholine, which is the most important neurotransmitter within the ENS in physiological conditions, loses its significance during processes occurring under the impact of pathological stimuli. Moreover, it is commonly known that acetylcholine is the main neuronal factor which increases intestinal motility [28,36]. Therefore, the decrease in the number of enteric cholinergic neurons noted under the impact of bisphenols, both in the present study and previous investigations [20,37], may be connected to the relatively well-known relaxant effect of BPA within the gastrointestinal tract [38]. On the other hand, the decrease in the number of cholinergic nerve structures in the wall of the gastrointestinal tract may be related to the direct effect of BPA on the nervous system and the neurotoxic effect of this substance, manifested by impaired development and function of synapses, as well as changes in neuroprotein expression and ion transport [39,40,41,42].

The obtained results have shown that the number of neurons immunoreactive to other neuronal factors studied increases under the impact of bisphenol A and S. This observation is in agreement with previous studies, in which the increase in the number of GAL-, SP-, VIP-, and/or nNOS-positive enteric neurons has been found under the impact of various pathological and toxic stimuli [30,31]. It is probably connected with the functions of the above-mentioned neuronal substances in the intestine, and therefore, a brief reminder of them seems justified.

It is well known that GAL plays a trophic role in the nervous system and protects neurons during neurodegenerative diseases such as Alzheimer’s and Parkinson’s [43]. In the gastrointestinal tract, in addition to neuroprotective properties [44,45], GAL regulates secretion, motility, blood flow, and immunological processes [30,46,47,48]. Therefore, the observed fluctuations in the number of GAL+ neurons may also be the result of BPA-induced disturbances in the functioning of the intestinal barrier, which has been described in previous investigations [25,37].

In turn, SP is mainly involved in the immunological reactions [49] and adaptive and protective processes taking place in the nervous tissue [50]. Therefore, the increase in the number of SP-immunoreactive neurons may result from the impact of bisphenols on the immune system and their relatively well-known pro-inflammatory activity. Although it is known that BPA not only affects the gastrointestinal mucosa, leading to increased apoptosis, inhibition of mucin secretion, and disruption of the intestinal barrier, it also causes an increase in the expression of pro-inflammatory cytokines [51]. On the other hand, SP is a key pro-inflammatory substance that has many important functions in the activation of the immune system, leading to an increase in the synthesis and expression of tumour necrosis factor-alpha and interleukins, including IL-1 and IL-6 [49]. Moreover, SP is involved in the regulation of intestinal motility [52], and the increase in the number of SP-positive cells under the impact of bisphenols may be the adaptive answer of the ENS on the decrease in the percentage of cholinergic neurons.

The next enteric neuronal populations included in this study are vipergic and nitrergic cells, which play various functions in the intestine. First of all, VIP and nitric oxide are the main inhibitory factors that cause the relaxation of intestinal smooth muscles [39]. Both VIP and NO are also antioxidants and factors that inhibit the secretion of the gastrointestinal tract [19,53]. In addition, these substances influence the intestinal blood flow, may affect immune reactions, and have relatively strong anti-inflammatory and neuroprotective properties [54,55,56,57]. Therefore, the increase in the number of VIP- and/or nNOS-immunoreactive neurons noted during the present study is probably related to the participation of these substances in adaptive and/or protective mechanisms occurring during exposure to bisphenols, as well as bisphenol-induced changes in the intestinal motility and proinflammatory activity of them.

In the case of BPS, similar changes were observed under the influence of a higher dose of this toxin. This may suggest that BPS is less toxic than BPA, but at a higher dose, it affects the organism in a way that is similar to BPA.

It should be noted that the impact of bisphenols on the digestive tract is multidirectional. These substances show not only neurotoxic and proinflammatory properties [51] and, as mentioned above, impair intestinal motility [58], but also the intestinal microbiome [59], which, in turn, may result in an increased risk of systemic toxicity [60]. It is also known that most colon diseases, such as ulcerative colitis, cancer, and aganglionosis, are accompanied by colonic motility disorders that may result from exposure to bisphenols [58,61,62]. This is confirmed by previous studies that showed a correlation between the severity of exposure to BPA and some pathological processes in the descending colon [63]. It should be emphasised that BPA not only affects the motility of the gastrointestinal tract but also increases the permeability of the mucosa by damaging the intestinal barrier [59], and also induces apoptosis and mitochondrial dysfunction in the gastro-intestinal mucosa layer, which is associated with BPA-induced oxidative stress and inflammatory processes [59,61]. All the effects of exposure to bisphenols mentioned above may be reflected in the changes in the neurochemical characteristics of enteric neurons observed in this study.

Moreover, the present study has shown that BPS has similar effects on the enteric neurons as BPA. This confirms previous studies that found that BPS is not safe for living organisms [3,10]. However, contrary to previous observations, which have described that BPS may have a stronger toxic effect than BPA [9], changes observed in the present study under the impact of BPS were visibly less severe than those noted under the impact of BPA. This may suggest that BPS (especially in lower doses) affects the ENS to a lesser extent than BPA.

Of course, the present study has some limitations. The most important of them is the fact that the immunofluorescence technique allows for changes in the number of neurons immunoreactive to particular substances occurring under the influence of BPA and BPS, but it does not explain the mechanisms underlying these changes. Therefore, further comprehensive research is necessary to elucidate all aspects of the effect of bisphenols on the ENS.

## 4. Material and Methods

To achieve the aim of the study, 35 CD1 strain mice (three months old at the start of the experiment, both genders) were used. The animals were divided into five groups (seven animals in each) according to the following scheme: C (control)—animals were not treated with bisphenols; BPA1—animals were treated with BPA in the dose of 5 mg/kg b.w./day; BPA2—animals received BPA at 50 mg/kg b.w./day; BPS1—animals were treated with BPS at 5 mg/kg b.w./day; and BPS2—animals received BPS at 50 mg/kg b.w./day.

The choice of the above-mentioned doses of bisphenols was because, based on previous studies, a BPA dose of 5 mg/kg b.w. has been described as a systemic no-observed-adverse-effect level (NOAEL) dose of this substance in CD1 mouse, and the dose of 50 mg/kg b.w. is a lowest-observed-adverse-effect level (LOAEL) dose for BPA in mouse [64,65,66].

All animals were kept under standard laboratory conditions, and bisphenols were given for three months in drinking water following the technique previously described by Makowska et al. 2023 [33]. All experimental procedures have been performed according to the approval of the Local Ethical Committee on the Experimental Animals in Olsztyn (Decision Nº 46/2019).

Immediately after euthanasia (by decapitation), 2 cm long parts of the jejunum (from the place located approx. 15 cm behind the stomach) were taken from every animal. Fragments of the intestine were put into 4% paraformaldehyde solution and fixed overnight. For three days, intestinal fragments were rinsed (at 4 °C) with everyday exchange of phosphate buffer (0.1 M, pH 7.4) and then put into 18% phosphate-buffered sucrose solution for at least three weeks at 4 °C.

**Figure 4:**
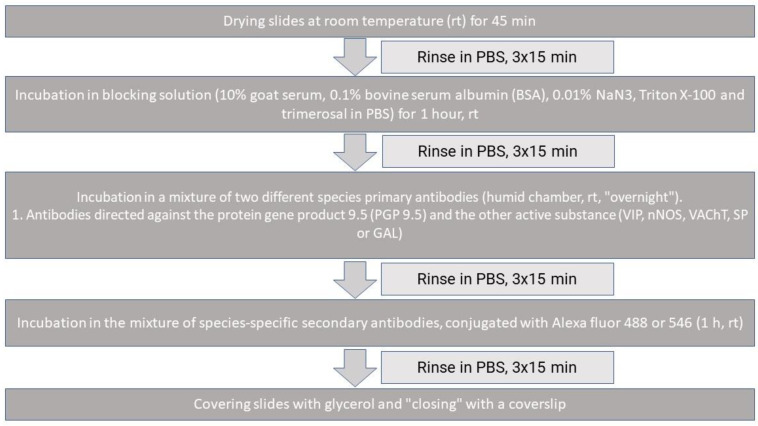
The immunofluorescence method.

Such prepared tissues were frozen at −20 °C, cut into 12 μm thick sections using a microtome (Microm, HM 525, Walldorf, Germany) and fixed on the microscopic slides. Intestinal sections were then subjected to the routine double immunofluorescence technique described previously by Wojtkiewicz et al. 2017 [67]. This method consists of several steps, as shown in Figure 4.

For the double immunofluorescence staining, commercial antibodies directed against protein gene product 9.5 (PGP 9.5—used here as a pan-neuronal marker), vasoactive intestinal polypeptide (VIP), galanin (GAL), substance P (SP), the neuronal isoform of nitric oxide synthase (nNOS—used as a marker of nitrergic markers), and vesicular acetylcholine transporter (VAChT—used as a marker of cholinergic neurons) were used. The exact specification of primary and secondary antibodies is presented in Table 3.

To exclude non-specific labelling, typical specificity tests (including pre-absorption of the antibodies with appropriate antigens and omission and replacement tests) were performed. Labelled tissues were evaluated with an epi-fluorescence microscope (Olympus, BX51), and the photographic documentation was made using a digital camera connected to a PC and processed with Olympus Cell F image analysis software version 1.17 (Olympus, Tokyo, Japan).

In determining the percentage of neurons immunoreactive to particular neuronal active substances, about 500 PGP 9.5-positive cells in each type of enteric plexus were assessed for each neuronal factor included in the study. The evaluation included only neurons with a visible cell nucleus, and the population of PGP 9.5 neurons was considered 100%. The obtained data were presented as the mean value ± SEM. In order to avoid double counting of the same cells, the sections evaluated were spaced at least 150 μm apart.

A statistical analysis of the obtained results was carried out with a one-way analysis of variance (ANOVA) with Dunnett’s test using Statistica 12 software (StatSoft Inc., Tulsa, OK, USA). Differences between studied groups were considered statistically significant at *p* < 0.05.

## 5. Conclusions

The present study is the first description of the influence of BPA and BPS administered in the same doses on the ENS in the mouse jejunum. The results show that the relatively low doses of both bisphenols used in the experiment do not cause any clinical symptoms but clearly influence the neurochemical characterisation of the enteric neurons. Therefore, changes observed in the ENS may be the first signs of the toxic activity of bisphenols and simultaneously the first answer of the gastrointestinal tract to exposure to these substances.

Comparing the influence of lower doses of BPA and BPS on the ENS, it has been found that BPA causes much more pronounced changes in the neurochemical characterisation of the enteric neurons, which might suggest that BPS is characterised by less toxicity and endocrine-disrupting activity than BPA. However, comparing the impact of higher doses of BPA and BPA, it has been proven that the administration of both bisphenols results in similar changes in the neurochemical characterisation of the enteric neurons. The observed similarities in the effects of BPA and BPS probably result from a similar chemical structure, as well as the similar endocrine-disrupting, pro-inflammatory, and neurotoxic properties of these substances.

The results have shown that although low doses of BPS affect the ENS to a lesser extent than BPA, this substance is not neutral for the nervous system supplying the GI tract, and exposure to it may result in changes in levels of neuronal active substances.

It should also be underlined that due to the multidirectional effects of bisphenols on the intestine and nervous system, the exact mechanisms of observed changes still remain unknown, and understanding these mechanisms requires further comprehensive research.

## Figures and Tables

**Table 1:** Partial results obtained in the experiment.

nNOS											
Group		C		BPA1		BPA2		BPS1		BPS2	
Ganglion		MP	SmP	MP	SmP	MP	SmP	MP	SmP	MP	SmP
Animal 1	A	506/200	498/141	505/209	508/174	504/307	475/167	501/216	503/178	506/215	500/194
	B	39.53	28.31	41.39	34.25	60.91	35.16	43.11	35.39	42.49	38.8
Animal 2	A	502/185	500/148	505/250	504/156	504/285	509/192	502/200	504/169	503/206	507/190
	B	36.85	29.6	49.5	30.95	56.55	37.72	39.84	33.53	40.95	37.47
Animal 3	A	502/200	502/147	502/201	508/174	512/235	503/194	503/206	502/178	509/214	505/199
	B	39.84	29.28	40.04	34.25	45.9	38.57	40.95	35.46	42.04	39.41
Animal 4	A	502/150	498/147	502/207	502/164	506/227	500/190	500/194	500/176	505/231	500/199
	B	29.88	28.31	41.24	32.67	44.86	38	38.8	35.2	45.74	39.8
Animal 5	A	509/196	498/150	503/194	505/135	502/242	502/201	505/200	503/185	509/225	502/200
	B	38.51	30.12	38.57	30.3	48.21	40.04	39.6	36.78	44.2	39.84
Animal 6	A	500/191	503/152	508/216	501/179	01/155	502/207	502/201	500/160	517/242	502/174
	B	38.2	30.22	42.52	35.73	30.94	41.24	40.04	32	46.81	34.66
Animal 7	A	509/193	505/156	512/235	500/152	501/216	512/235	507/201	504/174	504/269	501/167
	B	37.92	30.89	45.9	30.4	43.11	45.9	39.65	34.52	52.98	33.33
Total	C	**37.25** **± 1.28**	**29.53** **± 0.37**	**42.74** ± 1.42 *	**32.65** ± 0.82 *	**47.21** ± 3.67 *	**39.52** ± 1.29 *	**40.28** ± 0.53 *	**34.70** ± 0.58 *	**45.03** ± 1.54 *	**37.62** ± 0.99 *
**VIP**											
Animal 1	A	503/115	508/157	506/215	502/174	501/268	505/250	500/160	504/174	502/226	504/172
	B	22.86	30.91	42.49	34.66	53.49	49.5	32	34.52	45.02	34.13
Animal 2	A	500/134	501/148	503/206	502/170	503/194	506/140	508/164	502/152	499/200	502/155
	B	26.8	29.54	40.95	33.86	38.57	27.67	32.28	30.28	40.08	30.88
Animal 3	A	503/178	501/155	502/201	502/174	502/200	500/200	504/169	502/141	506/196	502/178
	B	35.39	30.94	40.04	34.66	39.84	40	33.53	28.09	38.74	35.46
Animal 4	A	501/179	500/143	502/207	509/196	502/200	501/201	507/161	500/160	506/208	498/165
	B	35.73	28.6	41.24	38.51	39.84	40.12	31.76	32	41.11	33.13
Animal 5	A	507/168	498/150	502/210	509/171	502/242	507/206	508/167	500/150	500/200	507/174
	B	33.14	30.12	41.83	33.6	48.21	40.63	32.87	30	40	34.32
Animal 6	A	502/147	502/144	498/177	502/176	504/267	509/214	498/150	501/152	485/190	504/150
	B	29.28	28.69	35.54	35.06	52.98	42.04	30.12	30.34	39.18	29.76
Animal 7	A	508/156	498/141	508/216	504/174	505/231	509/197	503/194	504/156	501/179	500/174
	B	30.71	28.31	42.52	34.52	45.74	38.7	38.57	30.95	35.73	34.8
Total	C	**30.56** **± 1.77**	**29.59** **± 0.42**	**40.66** ± 0.92 *	**34.98** ± 0.62 *	**45.52** ± 2.39 *	**39.81** ± 2.43 *	**33.02** **± 1.01**	**30.88** **± 0.75**	**39.98** ± 1.06 *	**33.21** ± 0.8 *
**GAL**											
Animal 1	A	504/134	500/110	509/128	509/114	503/178	500/143	509/128	509/122	500/143	500/120
	B	26.59	22	25.15	22.4	35.39	28.6	25.15	23.97	28.6	24
Animal 2	A	500/125	500/112	502/141	505/113	502/200	504/145	502/141	507/121	498/150	506/109
	B	25	22.4	28.09	22.38	39.84	28.77	28.09	23.86	30.12	21.54
Animal 3	A	502/144	500/104	504/156	507/127	502/150	499/166	500/134	504/117	500/176	505/112
	B	28.69	20.8	30.95	25.05	29.88	33.27	26.8	23.21	35.2	22.18
Animal 4	A	498/141	507/109	508/174	500/134	506/159	500/125	500/128	502/176	503/185	509/135
	B	28.31	21.5	34.25	26.8	31.42	25	25.6	35.06	36.78	26.52
Animal 5	A	505/120	487/101	502/141	504/141	501/174	498/150	507/139	487/101	508/164	504/141
	B	23.76	20.74	28.09	27.98	34.73	30.12	27.42	20.74	32.28	27.98
Animal 6	A	501/118	504/90	504/148	500/138	502/178	502/144	502/141	489/74	504/169	504/131
	B	23.55	17.86	29.37	27.6	35.46	28.69	28.09	15.13	33.53	25.99
Animal 7	A	498/114	500/79	502/147	506/151	507/197	503/155	504/156	500/79	500/120	502/109
	B	22.89	15.8	29.28	29.84	38.86	30.82	30.95	15.8	24	21.71
Total	C	**25.54** **± 0.89**	**20.16** **± 0.92**	**29.31** ± 1.06 *	**26.01** ± 1.08 *	**35.08** ± 1.36 *	**29.32** ± 0.96 *	**27.44** **± 0.73**	**22.54** **± 2.51**	**31.50** ± 1.64 *	**24.27** ± 0.98 *
**VAChT**											
Animal 1	A	500/250	502/242	500/242	500/196	514/241	502/199	504/269	508/216	503/235	506/208
	B	50	48.21	48.4	39.2	46.88	39.64	52.98	42.52	46.72	41.11
Animal 2	A	501/250	610/286	514/241	501/218	505/180	503/217	504/252	512/235	500/215	500/194
	B	49.9	46.88	46.88	43.51	35.64	43.14	50	45.9	43	38.8
Animal 3	A	548/279	548/279	505/180	503/215	505/182	510/200	505/241	506/227	507/220	507/190
	B	50.86	50.86	35.64	42.74	36.04	39.21	47.72	44.86	43.39	37.47
Animal 4	A	504/285	513/256	500/246	519/231	503/194	506/161	504/216	513/241	501/216	517/27
	B	56.55	49.9	49.2	44.51	38.57	31.82	42.86	46.98	43.11	40.04
Animal 5	A	500/250	501/280	500/250	505/190	519/231	511/225	500/250	510/213	502/200	507/201
	B	50	55.88	50	37.62	44.51	44.03	50	41.76	39.84	39.65
Animal 6	A	503/286	514/241	500/257	610/286	501/218	504/175	502/242	506/227	513/241	508/218
	B	56.86	46.88	51.4	46.88	43.51	34.72	48.21	44.86	46.98	42.91
Animal 7	A	509/307	503/227	502/230	503/230	508/216	502/201	500/256	503/250	503/235	508/216
	B	60.31	45.13	45.82	45.73	42.52	40.04	51.2	49.7	46.72	42.52
Total	C	**53.50** **± 1.63**	**49.11** **± 1.35**	**46.74** ± 1.98 *	**42.88** ± 1.27 *	**41.10** ± 1.62 *	**38.94** ± 1.65 *	**49.00** ± 1.22 *	**45.22** ± 1.01 *	**44.25** ± 1.01 *	**40.36** ± 0.74 *
**SP**											
Animal 1	A	489/74	505/101	509/105	500/125	505/182	507/174	487/101	507/109	509/128	507/122
	B	15.13	20	20.63	25	36.04	34.32	20.74	21.5	25.15	24.06
Animal 2	A	499/68	505/101	500/85	507/105	503/194	504/150	504/90	501/106	500/124	500/125
	B	13.63	20	17	20.71	38.57	29.76	1786	21.16	24.8	25
Animal 3	A	501/71	50/103	504/100	489/120	509/128	505/125	503/101	505/101	509/128	500/113
	B	14.17	20.6	19.84	24.53	25.15	24.75	20.08	20	25.15	22.6
Animal 4	A	487/101	500/97	507/109	500/113	502/141	500/141	500/89	505/101	505/21	500/114
	B	20.74	19.4	21.5	22.6	28.09	28.2	17.8	20	23.96	22.8
Animal 5	A	504/90	52/101	506/109	500/120	503/156	500/120	505/94	506/112	504/139	500/110
	B	17.86	20.12	21.54	24	31.01	24	18.61	20.16	27.58	22
Animal 6	A	500/99	498/93	500/99	500/113	506/135	509/128	503/116	500/106	507/109	509/122
	B	19.8	18.67	19.8	22.6	26.68	25.15	23.06	21.2	21.5	23.97
Animal 7	A	501/94	499/82	503/99	505/120	501/136	500/124	500/120	501/100	505/125	503/86
	B	18.76	16.43	19.68	23.76	27.15	24.8	24	19.96	24.75	17.1
Total	C	**17.16** **± 1.07**	**19.32** **± 0.53**	**20.00** ± 0.58 *	**23.31** ± 0.55 *	**30.38** ± 1.93 *	**27.28** ± 1.42 *	**20.15** ± 0.84 *	**21.15** ± 0.53 *	**24.70** ± 0.68 *	**22.50** ± 0.98 *

A—cell number PGP/other substance studied. B—% of cells immunoreactive to substance studied. C—mean % ± SEM of cells immunoreactive to substance studied. The results were considered statistically significant at *p* < 0.05 and are marked with *.

**Table 2:** The number of neurons immunoreactive to vasoactive intestinal polypeptide (VIP), neuronal isoform of nitric oxide synthase (nNOS), vesicular acetylcholine transporter (VAChT), substance P (SP), and galanin (GAL) in the myenteric plexus (MP) and submucous plexus (SmP) of mouse jejunum in the control group (C), after the administration of lower (BPA1) and higher (BPA2) dose of BPA and lower (BPS1) and higher (BPS2) dose of BPS. The results were considered statistically significant at *p* < 0.05 and are marked with *.

**nNOS**					
	**C**	**BPA1**	**BPA2**	**BPS1**	**BPS2**
MP	37.25 ± 1.28	42.74 ± 1.42 *	47.21 ± 3.67 *	40.28 ± 0.53 *	45.03 ± 1.54 *
SmP	29.53 ± 0.37	32.65 ± 0.82 *	39.52 ± 1.29 *	34.70 ± 0.58 *	37.62 ± 0.99 *
**VIP**					
	**C**	**BPA1**	**BPA2**	**BPS1**	**BPS2**
MP	30.56 ± 1.77	40.66 ± 0.92 *	45.52 ± 2.39 *	33.02 ± 1.01	39.98 ± 1.06 *
SmP	29.59 ± 0.42	34.98 ± 0.62 *	39.81 ± 2.43 *	30.88 ± 0.75	33.21 ± 0.8 *
**GAL**					
	**C**	**BPA1**	**BPA2**	**BPS1**	**BPS2**
MP	25.54 ± 0.89	29.31 ± 1.06 *	35.08 ± 1.36 *	27.44 ± 0.73	31.50 ± 1.64 *
SmP	20.16 ± 0.92	26.01 ± 1.08 *	29.32 ± 0.96 *	22.54 ± 2.51	24.27 ± 0.98 *
**VAChT**					
	**C**	**BPA1**	**BPA2**	**BPS1**	**BPS2**
MP	53.50 ± 1.63	46.74 ± 1.98 *	41.10 ± 1.62 *	49.00 ± 1.22 *	44.25 ± 1.01 *
SmP	49.11 ± 1.35	42.88 ± 1.27 *	38.94 ± 1.65 *	45.22 ± 1.01 *	40.36 ± 0.74 *
**SP**					
	**C**	**BPA1**	**BPA2**	**BPS1**	**BPS2**
MP	17.16 ± 1.07	20.00 ± 0.58 *	30.38 ± 1.93 *	20.15 ± 0.84 *	24.70 ± 0.68 *
SmP	19.32 ± 0.53	23.31 ± 0.55 *	27.28 ± 1.42 *	21.15 ± 0.53 *	22.50 ± 0.98 *

**Table 3:** The list of primary and secondary antibodies.

**Primary Antibodies**				
**Antigen**	**Code**	**Species**	**Working Dilution**	**Supplier**
PGP 9.5	7863-2004	Mouse	1:1000	BioRad, Hercules, CA, USA
VIP	11428	Rabbit	1:4000	Cappel, Aurora, OH, USA, 11428, working dilution 1:5000
SP	8450-0505	Rat	1:1000	BioRad
nNOS	AB5380	Rabbit	1:2000	Sigma-Aldrich, Saint Louis, MO, USA
GAL	T-5036	Guinea pig	1:2000	Peninsula Labs, San Carlos, CA, USA
VAChT	H-V006	Rabbit	1:2000	Phoenix Pharmaceuticals, Burlingame, CA, USA
**Secondary Antibodies Reagents**				
**Reagents**			**Working Dilution**	**Supplier**
Alexa fluor 488 donkey anti-mouse IgG			1:1000	ThermoFisher Scientific, Waltham, MA, USA
Alexa fluor 546 donkey anti-rabbit IgG			1:1000	ThermoFisher Scientific,
Alexa fluor 548 donkey anti-rat IgG			1:1000	ThermoFisher Scientific,
Alexa fluor 546 donkey anti-guinea pig IgG			1:1000	ThermoFisher Scientific,

## Data Availability

Data are contained within the article.
